# Fifty years of EPA science for air quality management and control

**DOI:** 10.1007/s00267-021-01468-9

**Published:** 2021-04-09

**Authors:** Charles Andrew Miller

**Affiliations:** U.S. Environmental Protection Agency, Office of Research and Development, Research Triangle Park, NC 27711 USA

**Keywords:** Air pollution, Air pollution control, Air quality modeling, Ambient air monitoring, History

## Abstract

Research and development has been a key part of the foundation for improvements in US air quality since the establishment of the Environmental Protection Agency (EPA) 50 years ago. Although the scientific accomplishments and advances over the course of EPA’s history are often overshadowed by policy debates, much of the air pollution science and engineering we now consider to be routine did not exist when EPA was established. Many of the advances in air pollutant measurement, monitoring, modeling, and control were developed by EPA researchers or supported by EPA programs. The technical foundation built during EPA’s early years has since given the Agency the scientific ability to respond quickly and effectively to unexpected and emerging issues. Equally important, EPA also developed approaches to conducting and presenting science in policy settings to ensure that the science was as objective and complete as possible and was communicated effectively. A look back at some of the accomplishments of EPA scientists and engineers provides a reminder that the cumulative effect of continual, incremental advances can result in large and lasting benefits to society.

## Introduction

In December 2020, EPA marked its 50th anniversary. There have been numerous activities to celebrate the work that the Agency has done over its half-century of existence, focusing on the impressive environmental improvements that have occurred as a result of EPA’s efforts. EPA’s science has been one of the aspects that enabled the Agency’s past and continuing success in fulfilling its mission. This anniversary provides an opportune time to recognize the solid and long-term foundation of science and engineering supporting EPA’s accomplishments.

Science and research have been at the heart of air pollution control since the first national legislation was passed in 1955. The 1955 Air Pollution Control Act was actually titled, “An Act to provide *research and technical assistance* relating to air pollution control” [emphasis added]. Fifteen years later, the Clean Air Act of 1970 further acknowledged the foundational role that research plays in the development and implementation of policy to improve and protect air quality. The first section of the 1970 Act, before any discussion of regulation or pollution control, begins, “The Administrator shall establish a national research and development program for the prevention and control of air pollution….” This language remains in the current law and describes what is required as part of the research program:

conduct, and promote the coordination and acceleration of, research, investigations, experiments, demonstrations, surveys, and studies relating to the causes, effects (including health and welfare effects), extent, prevention, and control of air pollution [42 USC 85, Part A, §7403; “including health and welfare effects” added in 1990].

The 1970 Act specifically called for research on air pollution monitoring, analysis, and modeling; on the short- and long-term health effects of air pollutants; and, in cooperation with the National Oceanographic and Atmospheric Administration, the Fish and Wildlife Service, and the U.S. Department of Agriculture, on the short- and long-term effects of air pollution on ecosystems.

The Act listed multiple activities that EPA was authorized to conduct as part of its national research and development program, including information dissemination, interagency cooperation, establishment of research grants and fellowships, and development of prototype air pollution control technologies.

This review is meant to provide a sense of the scope and importance of research to EPA’s ability to fulfill the mission it has been given by Congress to improve and maintain clean air. It is illustrative of the breadth of air-related research conducted by and for EPA over the past 50 years, focusing on work supported and conducted by EPA’s Office of Research and Development (ORD). This focus does not detract from critical research conducted and supported by other organizations, including the California Air Resources Board (2021). As is true for research in general, EPA’s air pollution research is characterized by a number of groundbreaking advances in knowledge and capabilities enabled and expanded upon by a much larger body of incremental improvements. These multi-faceted advances in science and technology enabled related advances in policies that have led to substantial, long-term improvements in air quality.

## Background

The volume of EPA’s research makes it impossible to cover the entire range of activities and accomplishments. Fortunately, there are some excellent discussions of EPA’s research in the literature for the reader interested in more focused descriptions of air quality research through the first half of EPA’s existence. Irwin’s history of air quality models provides a deep dive into the evolution of air quality models at EPA and elsewhere (Irwin 2004). Chow presents a thorough review of the evolution of air quality monitoring techniques for particulate matter (PM)(Chow 1995). Bachmann’s history of the National Ambient Air Quality Standards (NAAQS) includes discussions of several lines of research that informed the development of the Standards over the Agency’s history (Bachmann 2007).

Bachmann’s study also highlights a crucial point: the primary role of research in EPA is to inform, not determine, policy (Bachmann 2007). The difference between science and policy was also emphasized by Dowd and Yosie in their discussion of the role of science in the NAAQS development process (Dowd and Yosie 1981). The two must work together, and taking a long view of EPA’s accomplishments, it is clear that the partnership has been successful. But the partnership is not without its challenges. Even in the early days of EPA, it was understood that policy most often deals with the near term, which requires that research to inform policy have a near-term focus (Brown and Byerly 1981). Research can take months or years to come to conclusive results, building incrementally upon itself. EPA researchers balance the objectives of meeting near-term Agency needs while pursuing longer-term scientific development. It is this mix of near-term focus and insights into future issues that have made it possible for EPA research to address immediate needs while being prepared to respond to emerging issues.

A thorough examination of what EPA has contributed to environmental science for the development and implementation of policy could fill multiple books. There is a tremendous amount of EPA research on water quality, waste management, and general environmental quality that is beyond the scope of this paper. Even on the narrower topic of air quality, foundational work by EPA scientists on the health and environmental effects of air pollution (such as the early work of Schwartz on the link between PM and mortality (Schwartz 1991; Schwartz and Dockery 1992a, b)) must be set aside to provide meaningful detail on the research related to measurements, modeling, and control. Other key contributions from organizations such as research at the National Vehicle and Fuel Emissions Laboratory (Lindner and Schrodt 1993; Paulina 2004; Nam et al. 2010) and advances from EPA-supported research under the Science to Achieve Results (STAR) program are also recognized but cannot be covered here in the interest of brevity.

This discussion must also leave out the multitude of EPA’s international research interactions and collaborations on air quality management and control. From its earliest days, EPA recognized the importance of engaging with the international research community to exchange insights and advances (U.S. Environmental Protection Agency 1976). Such engagement ranged from exchanges at technical conferences (Chappell 1985) to technical support for full-scale demonstration (LaFlesh et al. 1993), and has continued through efforts such as support for air quality monitoring in US Embassies around the world.

The paper will begin with a brief discussion of the place of research in the Clean Air Act. It will then discuss research on air pollution control technologies, pollutant source and ambient measurements, air quality models, and an example of how these are combined to address a single regulatory issue. The paper will then provide a brief discussion of issues that have emerged since the founding of EPA, including global climate change. We will then look at the results of the work—what has the outcome of this research been? Finally, the paper will discuss the people who have planned and conducted the research and will close with a discussion of challenges.

## Pollution Control Technology Development

In 1970, our understanding of how pollutants were formed and how they could be controlled, especially in combustion processes, was far less developed than it is today. This is evident from the prominence given to air pollution control research in the 1955 Air Pollution Control Act and the 1970 Clean Air Act. EPA’s air pollution control research covered the development and evaluation of air pollution control technologies for stationary sources, from small industrial boilers to glass manufacturing to large coal-fired electricity generating units. These efforts covered the full range of research and development, from the fundamental chemistry and physics of pollutant formation through support for full-scale demonstrations of prototype technologies. In the early 1970s, the budgets for EPA research, including for energy research and development, accounted for as much as 30% of EPA’s operational budget (not including grants for construction of water treatment systems). By 1977, EPA’s energy research budget was equivalent to over $500 million in 2019 dollars (U.S. Environmental Protection Agency 1978), most of it to support research by experts in industry and academic institutions.

These investments have paid off well, providing the scientific foundation and technical basis for the science and technology we rely upon today to control sulfur dioxide (SO_2_), oxides of nitrogen (NO_*x*_), and PM from large electricity generating stations and industrial plants. Foundational research conducted and supported by EPA on technologies to control SO_2_, NO_*x*_, and PM and support for full-scale demonstrations of those technologies, led to the wide-spread deployment of commercialized versions of these systems. There is a direct line from EPA’s early control technology research and development to the commercial control technologies that have made it possible to achieve the substantial reductions in NO_*x*_ (U.S. Environmental Protection Agency 1980, 1995a), SO_2_ (U.S. Environmental Protection Agency 1995b), and PM (Lawless and Sparks 1980; Plaks and Sparks 1991) that have been achieved since the founding of the Agency.

A major accomplishment of EPA researchers was discovering the role of “fuel nitrogen” (i.e., nitrogen bound in the fuel) during the combustion of oil, synfuels, and pulverized coal (Beer et al. 1990). This fundamental finding enabled many of the subsequent NO_*x*_ control technologies developed by commercial firms for coal-fired boilers. Similarly, studies at EPA pilot-scale facilities produced several advances in SO_2_ scrubbers, including enhanced scrubber internal circulation, use of forced oxidation, and development of additives, all of which contributed to improved cost and operational performance. Lee (2013) documents how EPA researchers developed the technical capabilities to address the technical problems related to scrubber reliability (chemical scaling, plugging, erosion/corrosion, and mechanical problems). Perhaps as importantly, he describes how EPA researchers “shifted the focus from pollution abatement for health benefit to the development of pollution control technology for effective regulation.” This shift illustrated the recognition by EPA researchers that they needed to conduct their work in a way that indirectly advanced toward achieving the broader Agency mission: without effective regulation, the health benefits would not be realized. The technical advances focused on information needed to enable EPA to set technology-based emission standards, leading to health and environmental benefits and helping the advance ultimate worldwide commercial operation of SO_2_ controls (U.S. Environmental Protection Agency 1995b).

These early successes provided the experience that enabled EPA researchers to develop pollution control strategies that focused on operational practices, rather than requiring additional pollution control systems. In the mid-1980s, municipal waste combustors (MWCs), designed to burn municipal solid waste and generate electricity, and medical waste incinerators (MWIs) were found to be major emitters of chlorinated dioxins and furans. EPA’s expertise in combustion research was instrumental in identifying the chemical mechanisms that formed these compounds when chlorine-containing wastes were burned under conditions often found in MWC units. EPA engineers and scientists developed operating practices that formed much lower levels of dioxins and worked to make these “good combustion practices” the requirement for operating MWC and MWI systems (Kilgroe et al. 1992).

By working with the American Society of Mechanical Engineers, an operator training and certification program was developed (ASME 2020) that resulted in the reduction of dioxin emissions from MWC units by more than 99% between 1987 and 2000, relying on changes in the operation of existing equipment, and even as the number of MWC units more than doubled over the same time period (U.S. Environmental Protection Agency 2006).

The research experience gained in the development of large-scale pollution control systems was also applied to many smaller-scale controls. As awareness grew in the 1980s about the prevalence of residential exposure to radon from natural sources, EPA researchers developed techniques to reduce those exposures by preventing radon from entering buildings (U.S. Environmental Protection Agency 1994b) and guidance for builders and owners and operators of different building types (including homes and schools) about how to install these relatively simple techniques (Leovic and Craig 1994). One follow-up analysis found these measures reduced radon levels in sampled homes by more than 90% on average, which, if applied to all similar homes in Minnesota, was estimated to have the potential to extend the lives of 50,000 residents by nearly two decades (Steck 2012).

This evolution from conducting research and development on SO_2_ and NO_*x*_ controls for large utility and industrial plants to reducing dioxin from waste combustion and controlling radon in residences illustrates the long-term value of investing in core scientific and technical capabilities.

## Measurements

Among the least-well, recognized accomplishments of EPA’s scientific achievements is the development of the technical infrastructure required to reliably, repeatably, and accurately measure air pollutants. Measurements form the foundation of understanding air pollution in the atmosphere and the emissions of air pollutants from sources, both of which underlie effective air pollution policies. Since the establishment of EPA, thousands of studies have been prepared and published to explain how to extract samples of air or exhaust, analyze those samples for the presence of specific pollutants, and determine the quantity of those pollutants in the samples. While these efforts are nearly invisible to most people, they have made it possible to know not only the amount of pollution in the air but also the effectiveness of laws and policies to mitigate pollution.

Since its founding, scientists and engineers at EPA have contributed to the development, evaluation, and approval of nearly 200 official EPA methods for sampling and analyzing air pollutant emissions. In response to advances in technologies and changes in measurement needs, EPA has also evaluated and approved nearly 130 more alternative methods and identified 31 additional methods that may be appropriate for some situations (U.S. Environmental Protection Agency 2020a). The evaluation and approval process is extensive—one evaluation of procedures to evaluate just the airflow rate in one PM sampling system ran to 160 pages (Smith et al. 1978).

Approaches to measure emissions from sources without exhaust stacks differ significantly from those early methods developed to quantify stack emissions. EPA researchers were among the pioneers in applying one approach using remote emission measurement technologies. The use of open-path Fourier transform infrared (FTIR) spectroscopy to estimate emissions of methane from coal mines in the early 1990s is an example of these efforts (Kirchgessner et al. 1993). The FTIR technology was extended for use in measuring ammonia from hog waste lagoons (Harris et al. 2001). These and subsequent efforts were crucial to the acceptance of optical and remote sensing technologies for air pollutant measurement and monitoring, which are now recognized as important air quality management tools (U.S. Environmental Protection Agency 2018).

Measuring ambient concentrations used to determine air quality has its own challenges, including not just pollutant collection and quantification, but also the design of the monitoring network. Ambient monitoring of PM presents its own set of challenges. PM varies in size, shape, composition, density, and light absorption, among other parameters. Because it must be operationally defined, EPA’s early efforts to evaluate PM monitoring methods required testing a wide range of technologies, from the high volume air samplers (eventually chosen as the reference method) to visually determined opacity measures and tape-based sampling systems (Lee et al. 1972). These and other early efforts to measure PM at different sizes (Regan et al. 1979) eventually provided the information needed to show the serious health impacts associated with PM_2.5_ (PM with an aerodynamic diameter less than 2.5 μm). This early work and the continuing evaluation of monitoring systems to ensure reliable, accurate, and consistent measurements (Noble et al. 2001) provided a key component of the foundation for PM standards that are responsible for extending thousands of lives and saving the country billions of dollars (U.S. Environmental Protection Agency 1997a, 2011). The impacts extend beyond the U.S., as EPA’s methods are seen as the “gold standard” around the world for ensuring accuracy and reliability of air pollution measurement.

Beyond the development of specific methods is the development of the operational system that ensures measurements from one location or time are comparable to measurements at any other location or time. This has required the development of standards against which all instruments can be calibrated. For some pollutants, this takes the form of a standard calibration gas, prepared according to strict requirements developed by EPA in collaboration with what is now the National Institute of Standards and Technology (NIST; then the National Bureau of Standards). For other pollutants, such as ozone, a standard calibration gas is not possible because the gas is too reactive and will change during even a brief period of storage. In those cases, a system that generates a calibration standard for immediate use is required. EPA scientists developed the first Standard Reference Photometer in 1983, the design of which is now used by NIST as the primary national standard in the USA and used globally by the International Committee of Weights and Measures (Paur and McElroy 1979; NIST 2009).

## Modeling

Where measurements allow us to understand what has happened, computational models provide us with the crucial capability to gain insight into what can happen in the future by simulating how air pollutants react and flow through the atmosphere, including how they can be formed from precursor compounds.

From its early days, EPA scientists and engineers have worked to develop models that simulate the behavior of air pollutants. This work began with the development and evaluation of computational models that simulate how pollutants disperse from a source through the ambient air, depending upon how air flows act to stir the atmosphere at different altitudes and wind speeds (Holzworth 1972). Research to understand air pollutant dispersion also involved physical modeling. By 1974, EPA had constructed a wind tunnel to examine how pollutant dispersion was affected by different building characteristics (tall and thin vs. short and wide, for instance) (Snyder and Lawson 1976). These early dispersion studies provided the information needed to set exhaust stack height requirements to avoid excessive near-source pollutant levels in different situations. The approaches and capabilities developed by this work in the 1970s formed the foundation for later work, including evaluations of potential exposure to toxic compounds emitted during the destruction of the World Trade Center towers (Perry et al. 2004).

From the start of EPA in 1970, scientists were developing computational methods to simulate the fate and transport of airborne pollutants as evidenced by the plume dispersion techniques of Turner (Turner 1970) and the plume rise formulations of Briggs (1972). Soon afterward, research began on developing computational models that had the ability to simulate chemical reactions in the atmosphere, including reactions that were accelerated by exposure to sunlight. These models were critical to understanding how ozone was formed in the atmosphere and how it could be controlled. Beginning with simple “box models” (Schere and Demerjian 1977), this work evolved in the early 1980s toward more realistic simulations of atmospheric air currents and meteorology with pollutant sources in specific locations and increasingly complex chemical reactions (Lamb 1983). In the early days of air pollution modeling, EPA scientists needed to develop not only the mathematical expressions for the atmospheric chemistry and physics but also needed to develop an approach that would allow their computation by computer systems of the day in a way that would enable future expansion of the model as computational capabilities grew (Lamb 1984). By 1990, EPA’s contributions to air quality modeling made it possible for air quality models to be required by the Clean Air Act as a part of a state’s demonstration that it plans to meet the NAAQS for ozone (Irwin 2004).

## Bringing it Together to Control Mercury

In 2003, EPA proposed a regulation to reduce emissions of mercury from coal-fired power plants (U.S. Environmental Protection Agency 2004). The development of the regulation drew on EPA research covering numerous topics from source characterization to assessment of environmental and health impacts, illustrating the range of research needed to inform policy actions. Even though the rule addressed mercury as an air pollutant, the rulemaking took into account EPA research on the potential for health and environmental damage due to deposition of airborne mercury into water and soil (Crane 1992). Human exposure to mercury was also considered in developing the rule, including different factors that affect exposures, methods to model those exposures, and estimates of ingestion rates (Burns et al. 1982; U.S. Environmental Protection Agency 1997b; Swartout and Rice 2000). Policymakers relied on EPA’s air quality models to understand how mercury can be transported from a power plant to areas where it is deposited on the ground and in water, where people can be exposed to the mercury. These models also provided information on how the amount of mercury in soil and water in different locations could be reduced by controlling emissions at power plants (U.S. Environmental Protection Agency 1999). Accurate estimates of emissions, and ultimately of emission reductions, could only occur if accurate measurement methods existed. EPA researchers conducted extensive work to evaluate commercial emission monitoring systems and the calibration materials and methods to ensure those systems were accurate (ARCADIS 2005; Ryan 2005a; Ryan 2005b). At the same time, there was recognition that along with the need for measurement methods, emissions could only be reduced if we had effective and reliable technologies to achieve those reductions in practice. Again, EPA researchers provided information about control technologies for mercury (U.S. Environmental Protection Agency 2002b) and about how existing SO_2_ control technologies could provide some reduction in emissions (Srivastava 2000). Finally, the policymakers drew upon EPA research to understand how reductions in gaseous mercury could result in increased mercury levels in the ash and other residues remaining from coal combustion (Thorneloe 2005).

While this is not the end of the story, with legal challenges and policy shifts changing the regulatory outcome and considerable technology development supported by the U.S. Department of Energy (DOE) (Feeley et al. 2009), this example illustrates how EPA researchers contributed understanding of a wide range of issues that need to be considered in a major rulemaking for mercury control. In some cases, their work was targeted to specific questions and needs of the policymakers, and in others, research contributed more widely used tools and methods that eventually turned out to be of value to the development of this rule. This combination of focused research and development of the longer-term, broadly applicable capabilities that Brown and Byerly called “intellectual capital” (Brown and Byerly 1981) proved to be of substantial value to EPA’s efforts to reduce mercury in the environment.

## Applying Intellectual Capital to Respond to Emerging Issues and Emergencies

This intellectual capital, the accumulated knowledge and organizational capabilities built over decades of research and development, has provided EPA with the scientific and technical capacity to respond quickly to a broad range of emerging issues and unprecedented events that affect public health and the environment.

In 2001, the nation faced two unprecedented terrorist attacks, first on the World Trade Center in New York and the Pentagon in Washington, followed closely by efforts to expose people in media and government to anthrax spores. EPA played an important role in the national response, which was only possible because of the ability to draw up and apply the scientific understanding developed over time. In the immediate aftermath of the 9/11 attack and resulting collapse of the World Trade Center towers, EPA assisted with air quality monitoring to provide information about the hazards associated with the on-going emissions from the fires and dust from the towers’ collapse (Vette et al. 2004). EPA played a more prominent role in the decontamination of Congressional and Postal Service facilities affected by the anthrax attacks (Barth et al. 2003). A report on lessons learned from the anthrax response noted that “EPA responders had only limited technical information and experience for cleaning up biological contaminants. Despite these challenges, EPA was able to implement a plan of action based on sound science.” (U.S. Environmental Protection Agency 2002a).

As two additional examples, EPA’s expertise in combustion and combustion emissions was instrumental to emergency response efforts. The first was part of the Agency’s response to Hurricanes Katrina and Rita in 2005. EPA researchers evaluated the potential use of air curtain incinerators as a means to help dispose of the enormous volume of organic debris, such as downed trees and destroyed and demolished homes and buildings, created by Hurricane Katrina (Miller and Lemieux 2007). The second incident was the Deepwater Horizon oil spill. EPA scientists used balloon-based sampling systems to collect samples of smoke from oil burning on the surface of the ocean to evaluate the potential emissions of toxic compounds, including chlorinated dioxins and furans (Aurell and Gullett 2010).

This ability to refocus scientific and technical expertise, laboratory and testing facilities, and organizational infrastructure to new problems is being applied to today’s emerging issues, including the use of low-cost air pollution sensors, remediation of per- and polyfluoroalkyl substances, and wildfire smoke. But the most wide-ranging problem that has required (and continues to require) the ability to apply expertise in new ways is climate change.

When EPA was formed in 1970, the issue of human-driven climate change was still seen as a purely scientific concern with little immediate relevance to public policy. Just over 10 years later, EPA had begun to recognize climate change as a problem that would very likely require policy actions to meet the challenges of reducing greenhouse gases (GHGs) and adapting to the impacts of a warming climate (Seidel and Keyes 1983; Smith and Tirpak 1989).

By the 1990s, the scientific and technical intellectual capital built during the Agency’s first two decades was evident in EPA’s early actions to address climate change, including hosting a 1992 symposium on GHG emissions and mitigation (U.S. Environmental Protection Agency 1994a). EPA researchers presented information on a broad range of topics: GHG emission inventories, including sources of methane; geological carbon dioxide sequestration; use of solar and biomass energy; forest and agriculture as a carbon sink; and an overall evaluation of the technological challenges of CO_2_ reduction. EPA scientists also discussed our understanding of the interactions between ozone-depleting substances and climate change (Montzka et al. 2008; Newman et al. 2008).

EPA’s expertise on the sources, formation, and impacts of air pollution played a key role in the Agency’s 2009 determination that greenhouse gases represented an endangerment to human health and welfare. Their work showed that ozone concentrations would increase with a warming climate (U.S. Environmental Protection Agency 2009a), providing a key part of the scientific basis for the Agency’s determination that GHGs were pollutants under the provisions of the Clean Air Act (U.S. Environmental Protection Agency 2009b).

## Science-Policy Interface

At least as important as advances in air pollution science itself has been EPA’s formation of the process by which scientific understanding is transferred to Agency decision-making for the NAAQS. Congress required that the NAAQS be based on “the latest scientific knowledge” and left the details of how to determine that knowledge to EPA. The consequences of a wrong decision were substantial: setting a standard too high could leave millions of people vulnerable to the adverse effects of air pollution and setting a standard too low could cost millions of dollars without providing additional public health benefits. The process of setting the standard therefore needed to produce results that were not only solidly based on the latest scientific knowledge but were also trusted by stakeholders, including Congress, industries, and the public at large.

Working with input from the external Clean Air Science Advisory Committee (CASAC), EPA developed a process that included the assignment of dedicated staff to review the science and translate that science for use in policy decisions. Within the ORD, the Environmental Criteria Assessment Office was formed and given the responsibility to develop the synthesis and interpretation of the available science in what were known as Criteria Documents (CDs) (Bachmann 2007).

On the policy side, the Office of Air Quality Planning and Standards developed an “integrated assessment of the most critical policy-relevant information that was intended to bridge the gap between the CD and decisions required of the administrator” (Bachmann 2007). Throughout the process, external review was provided by experts on the independent CASAC, who reviewed not only the science and its policy-focused evaluation but also the process by which both were developed (Dowd and Yosie 1981).

The strength of the process was noted early during a lawsuit over the NAAQS for lead. As Bachmann (2007) noted:

The court also praised EPA’s execution of the expanded administrative procedures it had earlier required. It cited “the rigorous scientific and public review which permitted a thorough ventilation of the complex scientific and technical issues presented by this rulemaking proceeding.”

This basic approach, even as it was restructured in response to changing science and policy environments, has proven to be robust over the subsequent decades, despite continuing criticism and legal challenges.

## Outcomes

Determining the value of EPA’s research can be difficult at best. The value of a particular research effort is dependent on many factors that fall well outside the research domain. For air-related research, however, we have several strong indicators of the success and value of EPA’s air-related research that contributed to reductions in air pollution. The Office of Management and Budget (OMB) estimated the annual net benefit of EPA’s air regulations to be between $123 billion and $640 billion (in 2016 dollars) for the period October 2006 through September 2016 (Office of Management and Budget 2017). EPA prepared two reports on the benefits and costs of the Clean Air Act. The first of these estimated a total net benefit to the U.S. of $40.3 trillion dollars (in 2016 dollars; $21.7 trillion in 1990 dollars) between 1970 and 1990 (U.S. Environmental Protection Agency 1997a). The second report covered the period 1990 to 2020, and estimated a total net benefit of $13.9 trillion (in 2016 dollars; $12 trillion in 2006 dollars) (U.S. Environmental Protection Agency 2011). This is an average of $460 billion per year over the 30-year period, consistent with OMB’s annual estimated net benefits. The total estimated net benefit of $54.2 trillion compares to a total national expenditure on energy of $47.3 trillion (2016 dollars) between 1970 and 2017, as estimated by the Energy Information Administration (EIA 2020). If the EPA net benefit estimates are correct (and these values are the *central* estimates of net benefits), implementing the Clean Air Act in essence covered the nation’s entire energy bill since 1970.

How much of these savings can be directly attributed to EPA’s research is considerably less certain. What is perhaps more certain is that EPA’s accomplishments could not have been as successful over such a long period of time without the scientific and technical information upon which the regulations have been based. Between 1970 and 2018, the nation invested just over $37 billion (2016 dollars) in EPA’s ORD (U.S. Environmental Protection Agency 2020b). Only a portion of that investment was for air-related research, and only a portion of the net benefits can be attributed to scientific and technological advances. But if even 1% of the net benefits can be attributed to research accomplishments, that is still a benefit of more than 10 times the total investment in research for all media.

It must be recognized that not all of EPA’s research has been successful. Research inherently involves work that does not result in meaningful advances. In hindsight, we can see instances in which research is perhaps halted prematurely, only to be seen later as important. For example, it is possible to consider an alternative scenario in which a few changes to research efforts in the late 1970s might have identified PM_2.5_ as a major pollutant well before 1997 (Bachmann 2007).

There are also instances in which EPA research followed a path that was ultimately unsuccessful as measured by ultimate application in practice, despite considerable initial success. Reburning, or multi-stage fuel injection, for control of NO_*x*_ from coal-fired furnaces, is a good example. Jointly with the Gas Research Institute and the Department of Energy, EPA research and development on reburning was recognized with a national award for advances in environmental technology from the Air & Waste Management Association in 1997 (Air & Waste Management Association 2021), but is no longer used as a significant means of NO_*x*_ control in the USA.

The realities of research include efforts that fail in the moment only to be of value later, those that fail altogether, fruitful research to inform policies that are ultimately changed, and research that identifies ineffective technical or policy approaches that enable decision-makers to avoid wrong decisions. These outcomes are an integral part of a vibrant research effort but are impossible to objectively quantify, even in hindsight. The preceding evaluation of the estimated economic costs and benefits is an effort to illustrate that, over the long term, the successes of EPA’s research have far outweighed investments that were not successful.

**Fig. 1:**
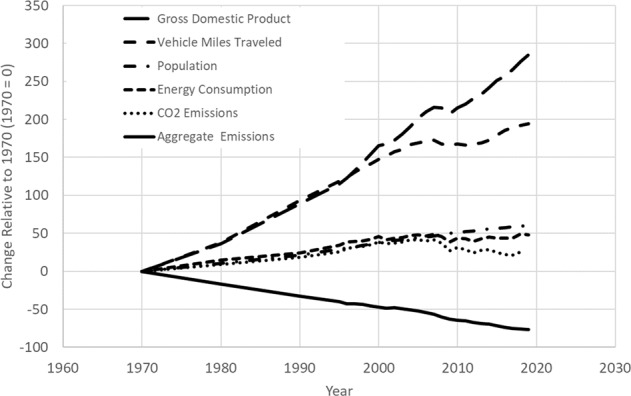
Comparison of growth areas and declining emissions, 1970–2018. Aggregate emissions are the combined emissions of PM_2.5_ and PM_10_, SO_2_, NO_x_, VOCs, CO, and Pb. From Our Nation’s Air (U.S. Environmental Protection Agency 2020c)

The impact of EPA’s science-based policies is evident from the trends in emissions from 1970 to 2018 and compared to key social and economic indicators, as seen in Fig. 1.

## People

None of these accomplishments could have been possible without a dedicated and talented staff of scientists, engineers, and technicians, and equally dedicated and talented technical and administrative staff to support them, and research and technical management to provide guidance and leadership. Only once in EPA’s 50 years has the ORD had more than 2000 full-time equivalent employees. As a comparison, DOE’s 17 National Laboratories have nearly 64,000 employees (National Laboratories Directors Council 2020).

An indication of the capabilities of EPA researchers studying air pollution issues is illustrated in the extent to which they have been recognized within the Agency and by other organizations. They have received Presidential-level awards, such as the Presidential Early Career Award for Scientists and Engineers. Air researchers have been named as finalists for the Samuel J. Heyman Service to America awards (the “Sammies,” often referred to as the Oscars of government service). They have been recognized by, and held national leadership roles in, professional societies such as the American Thoracic Society, the Air & Waste Management Association, and the American Society of Mechanical Engineers. They have been recognized within EPA by Gold Medals for Exceptional Service and by Scientific and Technological Achievement Awards, determined by EPA’s external Science Advisory Board.

Research at EPA is different than research at an academic institution. As one long-time EPA researcher, R.G. Lamb, noted in the Preface to his 1984 report on air quality modeling (Lamb 1984):

In attempting to use science as a tool for treating the types of applied problems that are of concern to the EPA, one is not allowed the luxury of simplifying assumptions that reduce problems to forms that possess concise elegant solutions. Instead, one must face the harsh realities of the physical world and search for approximate descriptions of phenomena that strike an acceptable compromise between rigor and practicability.

The record of scientific achievement and environmental improvement over the last 50 years shows that the people at EPA have been successful in striking that acceptable compromise.

And the Agency has expanded the nation’s scientific and technical expertise well beyond the halls of EPA. Research grants from the STAR program have supported the education and training of countless graduate students in the environmental sciences. EPA has awarded hundreds, if not thousands, of post-doctoral fellowships for recent science and engineering doctoral graduates to work in EPA laboratories. Many of these researchers gain valuable experience addressing real-world environmental problems before they take positions as faculty in colleges and universities throughout the country. EPA researchers engage with students at all levels to encourage interest in the environment and science, technology, engineering, and mathematics (STEM) studies more generally. These efforts show that EPA’s people continue working to build the expertise that will be needed for the future.

## Remaining Challenges

Because EPA science is necessarily a compromise between rigor and practicability, and because the science is intended to provide the basis for important policy decisions, the work of EPA researchers is generally subject to much more frequent and intense scrutiny than that of researchers in the academic community. EPA’s research can have enormous implications for industries and the public, who need to know that the results are accurate, repeatable, and robust. To ensure that EPA’s research activities and results are suitable for use in such consequential applications, they are subject to review and guidance by numerous external groups, including the National Academies of Sciences, Engineering, and Medicine; EPA’s external Science Advisory Board, Clean Air Science Advisory Committee, and Board of Scientific Counselors; the Government Accountability Office; and Congress. In addition to these reviews, EPA’s research is also subject to the requirements of the Information Quality Act and data quality and accessibility guidelines from the Office of Management and Budget. The efforts and resources in time and money needed to meet these requirements are rarely, if ever, seen by the general public. But they are crucial component to ensuring that EPA’s research can be trusted. Building and maintaining this trust is one of the core reasons why EPA’s research has been successful over the past 50 years.

Forty years ago Dowd and Yosie (1981) stated that “Research, as conducted by a regulatory agency, is an extremely fragile undertaking.” Their experiences, Yoshie as the federal official who coordinated the activities of CASAC and Dowd as an acting head of EPA’s ORD, allowed them to experience the unique challenges of conducting research for supporting public policy. They recognized that there are fundamental differences between researchers and policymakers that create tensions, even when the two groups have the same overarching goals:

In general, [EPA’s regulatory] program offices have been able to direct the research agenda…by demonstrating a need for short-term studies to support a particular standard-setting action. At times, conflict between ORD and program offices over research priorities has resulted in reductions of ORD’s budget and personnel ceilings after the priorities were ranked in the budgetary process. If the research committees’ focus is solely on short-term results, however, development of a scientific database for standard setting may be inhibited in such fundamental areas as atmospheric chemistry, chronic exposure to pollutants, and population studies.

They noted the challenge to sustaining the intellectual capital needed to maintain EPA science in a position to respond to the Agency’s evolving needs: “The infrastructure for maintaining a high-quality research staff is extremely sensitive to these constantly changing decisions on research priorities and budgets” (Dowd and Yosie 1981).

These challenges were echoed by Brown and Byerly (1981) writing at the same time as Dowd and Yosie (1981), but from a different perspective—that of Congress. They quoted Henry Kissinger’s 1959 commentary in The Reporter:

The administrative approach to intellectual effort tends to destroy the environment from which innovation grows. Its insistence on “results” discourages the intellectual climate that might produce important ideas whether or not the bureaucracy feels it needs them.

They discussed multiple institutional challenges to research in EPA, many of which remain four decades after their comments: finding the right balance between research to address immediate agency needs and longer-term research to build intellectual capital, dealing with limited resources and seemingly unlimited problems, and bridging the gap between scientists and policymakers. These challenges have been, and continue to be, the subject of efforts to plan and conduct research within EPA.

And yet, it is clear that EPA’s science-based policies have been spectacularly successful over its 50 years of existence. It may well be that the challenges of conducting and using research in a regulatory agency are both inherent and necessary. Indeed, it may be that those challenges and tensions push researchers toward practical, near-term results and policymakers toward consideration of longer-term questions, both of which can help EPA build on its record of success for the next 50 years.

## References

[CR1] Air & Waste Management Association (2021) J. Deane Sensenbaugh Environmental Technology Award. Air & Waste Management Association, https://www.awma.org/content.asp?contentid=403. Accessed 24 Mar 2021

[CR2] ARCADIS (2005). Long-Term Field Evaluation of Mercury Continuous Emission Monitoring System: Coal-fired Power Plant Equipped with Fabric Filtration/Wet Flue Gas Desulfurization Emission Controls.

[CR3] ASME (2020) QRO - Certification for Municipal Solid Waste Combustion Facilities Operator. ASME https://www.asme.org/certification-accreditation/personnel-certification/qro-certification-for-municipal-solid-waste-combustion-facilities-operator. Accessed 30 July 2020

[CR4] Aurell J, Gullett B (2010). Aerostat sampling of PCDD/PCDF emissions from the Gulf oil spill in situ burns. Environ Sci Technol.

[CR5] Bachmann J (2007). Will the circle be unbroken: a history of the U.S. National Ambient Air Quality Standards. J Air Waste Manag.

[CR6] Barth E, Rupert R, Stroud F, Rice E, Potoka B (2003). Environmental response to intentional dissemination of Bacillus anthracis spores in the United States–2001. Rem J.

[CR7] Beer JM, Bowman CT, Chen SL, Corley TL, De Soete GG (1990). Pulverized Coal Combustion: Pollutant Formation and Control, 1970-1980.

[CR8] Briggs GA (1972). Chimney plumes in neutral and stable surroundings. Atmos Environ.

[CR9] Brown G, Byerly R (1981). Research in EPA: a congressional point of view. Science.

[CR10] Burns LA, Cline DM, Lassiter RR (1982). Exposure Analysis Modeling System (EXAMS): user manual and system documentation.

[CR11] California Air Resources Board (2021) History. California Air Resources Board https://ww2.arb.ca.gov/about/history. Accessed 27 Jan 2021

[CR12] Chappell JP (1985). Project summary: proceedings: first joint symposium on dry SO2 and simultaneous SO2/NOx control technologies.

[CR13] Chow JC (1995). Measurement methods to determine compliance with ambient air quality standards for suspended particles. J Air Waste Manag.

[CR14] Crane JL (1992). Assessment and Remediation of Contaminated Sediments (ARCS) program: baseline human health risk assessment: Saginaw, Michigan, area of concern.

[CR15] Dowd RM, Yosie TF (1981). The role of science in EPA decision making. Environ Sci Technol.

[CR16] EIA (2020) January 2020 Monthly Energy Review. U.S. Department of Energy, Energy Information Administration, Washington, DC

[CR17] Feeley TJ, Jones AP, Brickett LA, O’Palko BA, Miller CE, Murphy JT (2009). An update on DOE’s Phase II and Phase III mercury control technology R&D program. Fuel Process Technol.

[CR18] Harris DB, Thompson EL, Vogel CA, Hashmonay RA, Natschke DF and Wagoner K (2001) Innovative Approach for Measuring Ammonia and Methane Fluxes from a Hog Farm Using Open-Path Fourier Transform Infrared Spectroscopy. Paper presented at the Air & Waste Management Association Annual Meeting. Orlando, FL

[CR19] Holzworth GC (1972). Mixing heights, wind speeds, and potential for urban air pollution throughout the contiguous United States.

[CR20] Irwin JS (2004) A historical look at the development of regulatory air quality models for the United States Environmental Protection Agency. In: Zannetti P (ed) Air quality modeling - theories, methodologies, computational techniques, and available databases and software. Vol. II – advanced topics. The EnviroComp Institute and the Air & Waste Management Association, Pittsburgh, PA

[CR21] Kilgroe JD, Lanier WS, von Alten TR (1992) Development of good combustion practices for municipal waste combustors. Paper presented at the 15th National ASME Waste Processing Conference. Detroit, MI

[CR22] Kirchgessner DA, Piccot SD, Chadha A (1993). Estimation of methane emissions from a surface coal mine using open-path FTIR spectroscopy and modeling techniques. Chemosphere.

[CR23] LaFlesh RC, Lewis RD, Hall RE, Kotler VR, Mospan YM (1993) Three-stage combustion (reburning) test results from a 300-MWe boiler in the Ukraine. Paper presented at the Environmental Protection Agency/Electric Power Research Institute Symposium on Stationary NOx Control. Miami, FL

[CR24] Lamb RG (1983). A regional scale (1000 km) model of photochemical air pollution. Part 1. Theoretical formulation.

[CR25] Lamb RG (1984). A regional scale (1000 km) model of photochemical air pollution. Part 2. Input Processor Network Design.

[CR26] Lawless PA, Sparks LE (1980). A mathematical model for calculating effects of back corona in wire‐duct electrostatic precipitators. J Appl Phys.

[CR27] Lee J (2013). Engineering the environment: regulatory engineering at the U.S. Environmental Protection Agency, 1970-1980. Dissertation, Virginia Polytechnic Institute and State University

[CR28] Lee RE, Caldwell JS, Morgan GB (1972). The evaluation of methods for measuring suspended particulates in air. Atmos Environ.

[CR29] Leovic KW, Craig AB (1994). Radon prevention in the design and construction of schools and other large buildings.

[CR30] Lindner J, Schrodt T (1993) Use of a repeatable car to improve intra-lab variability of emissions tests. Paper presented at the SAE International Congress and Exposition. Detroit, MI

[CR31] Miller C, Lemieux P (2007). Emissions from the burning of vegetative debris in air curtain destructors. J Air Waste Manag.

[CR32] Montzka SA, Daniel JS, Cohen J, Vick K (2008) Current trends, mixing ratios, and emissions of ozone-depleting substances and their substitutes.In: Ravishankara AR, Kurylo MJ, Ennis CA (eds) Trends in emissions of ozone-depleting substances, ozone layer recovery, and implications for ultraviolet radiation exposure. National Climatic Data Center, Asheville, NC

[CR33] Nam E, Kishan S, Baldauf RW, Fulper CR, Sabisch M, Warila J (2010). Temperature effects on particulate matter emissions from light-duty, gasoline-powered motor vehicles. Environ Sci Technol.

[CR34] National Laboratories Directors Council (2020) The National Laboratories: staff. National Laboratories Directors Council https://nationallabs.org/staff/. Accessed 24 Feb 2020

[CR35] Newman PA, Herman JR, Bevilacqua R, Stolarski R, Keating T (2008) Ozone and UV observations.In: Ravishankara AR, Kurylo MJ and Ennis CA (eds) Trends in emissions of ozone-depleting substances, ozone layer recovery, and implications for ultraviolet radiation exposure. A Report by the U.S. Climate Change Science Program and the Subcommittee on Global Change Research. National Climatic Data Center, Asheville, NC

[CR36] NIST (2009) The NIST standard reference photometer for ozone measurement traceability. National Institute of Standards and Technology https://www.nist.gov/programs-projects/nist-standard-reference-photometer-ozone-measurement-traceability. Accessed 30 July 2020

[CR37] Noble CA, Vanderpool RW, Peters TM, McElroy FF, Gemmill DB, Wiener RW (2001). Federal reference and equivalent methods for measuring fine particulate matter. Aerosol Sci Technol.

[CR38] Office of Management and Budget (2017). Draft report to congress on the benefits and costs of federal regulations and agency compliance with the unfunded mandates reform act.

[CR39] Paulina CM (2004). Hydrogen fuel cell vehicle fuel economy testing at the U.S. EPA National Vehicle and Fuel Emissions Laboratory. SAE Trans.

[CR40] Paur RJ, McElroy FF (1979). Technical assistance document for the calibration of ambient ozone monitors.

[CR41] Perry SG, Heist DK, Thompson RS, Synder WH, Lawson RE, Jr. (2004) Wind tunnel simulation of flow and pollutant dispersal around the World Trade Center site. EM: AWMA Magazine

[CR42] Plaks N, Sparks LE (1991) Electroprecipitator with alternating charging and short collector sections. Patent 5,059,219, U.S. Patent and Trademark Office

[CR43] Regan GF, Goranson SK, Larson LL (1979). Use of tape samplers as fine particulate monitors. J Air Poll Contr Assoc.

[CR44] Ryan JV (2005). Field Test Plan for Collection of Data to Support Development of an Instrumental Reference Method for Gaseous Mercury Emissions.

[CR45] Ryan JV (2005). March 10, 2005 Memorandum to B. Maxwell, results of recent testing of oxidized Hg Gas standards.

[CR46] Schere KL and Demerjian K (1977) A photochemical box model for urban air quality simulation. In Proceedings: 4th Joint Conference on Sensing of Environmental Pollutants. American Chemical Society, New Orleans, LA, pp. 427–433

[CR47] Schwartz J (1991). Particulate air pollution and daily mortality in Detroit. Environ Res.

[CR48] Schwartz J, Dockery DW (1992). Increased mortality in Philadelphia associated with daily air pollution concentrations. Am Rev Respir Dis.

[CR49] Schwartz J, Dockery DW (1992). Particulate air pollution and daily mortality in Steubenville, Ohio.. Am J Epidemiol.

[CR50] Seidel S, Keyes D (1983). Can We delay a greenhouse warming?.

[CR51] Smith F, Wohlschlegel PS, Rogers RSC, Mulligan D (1978). Investigation of flow rate calibration procedures associated with the high volume method for determination of suspended particles.

[CR52] Smith JB, Tirpak DA (1989). The potential effects of global climate change on the United States.

[CR53] Snyder WH, Lawson RE (1976). Determination of a necessary height for a stack close to a building—a wind tunnel study. Atmos Environ.

[CR54] Srivastava RK (2000). Controlling SO2 emissions: a review of technologies.

[CR55] Steck DJ (2012). The effectiveness of mitigation for reducing radon risk in single-family Minnesota homes. Health Phys.

[CR56] Swartout J, Rice G (2000). Uncertainty analysis of the estimated ingestion rates used to derive the methylmercury reference dose. Drug Chem Toxicol.

[CR57] Thorneloe SA (2005) February 18, 2005 Memorandum to S.L. Shaver, potential for cross-media transfers from management of mercury-enriched coal combustion residues. US EPA Office of Research and Development, Research Triangle Park, NC

[CR58] Turner DB (1970). Workbook of atmospheric dispersion estimates.

[CR59] U.S. Environmental Protection Agency (1976). Summmaries of foreign government environmental reports.

[CR60] U.S. Environmental Protection Agency (1978). Fiscal year 1978 budget.

[CR61] U.S. Environmental Protection Agency (1980). Controlling nitrogen oxides.

[CR62] U.S. Environmental Protection Agency (1994). Proceedings: the 1992 greenhouse gas emissions and mitigation research symposium.

[CR63] U.S. Environmental Protection Agency (1994). Radon mitigation research.

[CR64] U.S. Environmental Protection Agency (1995). Combustion modification control of nitrogen oxides.

[CR65] U.S. Environmental Protection Agency (1995). Flue gas desulfurization technologies for control of sulfur oxides: research, development, and demonstration.

[CR66] U.S. Environmental Protection Agency (1997). The benefits and costs of the clean air act, 1970 to 1990.

[CR67] U.S. Environmental Protection Agency (1997). Exposure factors handbook.

[CR68] U.S. Environmental Protection Agency (1999). User manual for the EPA third-generation air quality modeling system (Models-3 Version 3.0).

[CR69] U.S. Environmental Protection Agency (2002). Challenges faced during the Environmental Protection Agency’s response to anthrax and recommendations for enhanclng response capabllltles: a lessons learned report.

[CR70] U.S. Environmental Protection Agency (2002). Control of mercury emissions from coal-fired electric utility boilers.

[CR71] U.S. Environmental Protection Agency (2004) Proposed national emission standards for hazardous air pollutants; and, in the alternative, proposed standards of performance for new and existing stationary sources: electric utility steam generating units. Fed Register 69:4651–4752

[CR72] U.S. Environmental Protection Agency (2006). An inventory of sources and environmental releases of dioxin-like compounds in the United States for the Years 1987, 1995, and 2000.

[CR73] U.S. Environmental Protection Agency (2009). Assessment of the impacts of global change on regional U.S. air quality: a synthesis of climate change impacts on ground-level ozone.

[CR74] U.S. Environmental Protection Agency (2009). Endangerment and cause or contribute findings for greenhouse gases under section 202(a) of the clean air act. Fed Register.

[CR75] U.S. Environmental Protection Agency (2011). The benefits and costs of the clean air act from 1990 to 2020.

[CR76] U.S. Environmental Protection Agency (2018). EPA handbook: optical and remote sensing for measurement and monitoring of emissions flux of gases and particulate matter.

[CR77] U.S. Environmental Protection Agency (2020a) Air Emission Measurement Center (EMC). U.S. Environmental Protection Agency https://www.epa.gov/emc. Accessed 13 Aug 2020

[CR78] U.S. Environmental Protection Agency (2020b) Historical planning, budget, and results reports. U.S. Environmental Protection Agency. Accessed 6 Feb 2020

[CR79] U.S. Environmental Protection Agency (2020c) Our nation’s air. U.S. Environmental Protection Agency https://gispub.epa.gov/air/trendsreport/2020/. Accessed 13 Nov 2020

[CR80] Vette A, Seila R, Swartz E, Pleil J, Webb L, Landis M, Huber A and Vallero D (2004) Air pollution measurements in the vicinity of the World Trade Center. EM: AWMA

